# GPs’ patterns of clinical assessment when faced with a patient suspected for spondyloarthritis: a prospective educational intervention study

**DOI:** 10.3399/bjgpopen17X100689

**Published:** 2017-01-09

**Authors:** Marloes van Onna, Simone Gorter, Bas Maiburg, Gerrie Waagenaar, Astrid van Tubergen

**Affiliations:** 1 Rheumatologist, Department of Medicine, Division of Rheumatology, Maastricht University Medical Center, and School for Public Health and Primary Care (CAPHRI), Maastricht University Medical Center, Maastricht, the Netherlands; 2 Rheumatologist, Department of Medicine, Division of Rheumatology, Maastricht University Medical Center, and School for Public Health and Primary Care (CAPHRI), Maastricht University Medical Center, Maastricht, the Netherlands; 3 GP, Department of General Practice, Maastricht University, Maastricht, the Netherlands; 4 GP, Department of General Practice, Maastricht University, Maastricht, the Netherlands; 5 Rheumatologist, Department of Medicine, Division of Rheumatology, Maastricht University Medical Center, and School for Public Health and Primary Care (CAPHRI), Maastricht University Medical Center, Maastricht, the Netherlands

**Keywords:** general practice, medical education, spondyloarthritis

## Abstract

**Background:**

Timely recognition and referral of patients with spondyloarthritis (SpA) is challenging due to the frequent unawareness of the clinical picture.

**Aim:**

To identify clinical assessment patterns of GPs and GP-residents when facing a patient suspected of having SpA, and to determine which components of clinical assessment were most prevalent prior to referral to the rheumatologist and whether targeted education could positively influence pattern recognition.

**Design & setting:**

Prospective multicentre educational intervention study in primary care practices in the Netherlands.

**Method:**

GPs and GP-residents were visited in two rounds by standardised patients (SPs) simulating axial or peripheral SpA (dactylitis). Between these rounds, an educational intervention regarding SpA took place for part of the participants. SPs completed a case-specific checklist inquiring about disease-related items and items on physical examination.

**Results:**

Sixty-eight participants (30 GPs and 38 GP-residents) were included and 19 (28%) received the educational intervention. In round 1, about half of the participants asked at least one question to differentiate between an inflammatory or mechanical origin of the back pain or peripheral complaint; on average, <15% asked for extra-articular manifestations. After education, GP-residents inquired more about the presence of extra-articular manifestations and family history of axial SpA; this pattern was also observed in the GPs and GP-residents who correctly referred the SP. In the peripheral SpA case, the observed gain was less evident when compared to the axial SpA case.

**Conclusion:**

Pattern recognition of patients suspected for SpA by GP(-residents) is essential for referral to a rheumatologist and can be improved by education.

## How this fits in

Spondyloarthritis is often underrecognised in primary care, which contributes to a delay in diagnosis. Pattern recognition of patients suspected for spondyloarthritis is essential for referral to a rheumatologist. For correct pattern recognition, clinical skills such as history taking and physical examination are essential. Improving clinical skills by providing education contributes to correct pattern recognition and may therefore support the effective implementation of a referral strategy.

## Introduction

Musculoskeletal complaints account for 15–20% of all consultations in primary care but are often underemphasised in the GP residency training.^1,2^ This may result in an inadequate filtering of those patients with a high suspicion of an inflammatory rheumatic disorder and subsequently to a diagnostic delay.^3^ In the case of axial spondyloarthritis (axSpA), the diagnostic delay may be up to 10 years or longer, which may in turn lead to unnecessary diagnostic procedures and initiation of ineffective treatment by other care providers in the period prior to diagnosis.^4–7^

To facilitate a timely diagnosis of axSpA, the Assessment of SpondyloArthritis international Society (ASAS) has formulated a referral recommendation for patients suspected of having axSpA.^8^ In this recommendation, referral is advised for patients with chronic back pain (duration ≥3 months) which started before the age of 45 years in combination with at least one other relevant SpA feature; for example, inflammatory back pain (IBP), human leukocyte antigen (HLA)-B27 positivity, or the presence of an extra-articular manifestation.^8^

For correct use of the ASAS referral tool, clinical skills such as history taking and physical examination are essential to identify disease patterns that may raise suspicion on SpA. However, earlier studies have shown that the knowledge level of GPs about important clinical manifestations of SpA is inadequate,^3,9^ but that an educational programme targeted at recognition of SpA clearly improves referral of patients suspected for axial or peripheral SpA.^10^ The aim of the present study was to identify clinical assessment patterns of GP(-residents) when facing a patient suspected of having SpA, and to determine which components of clinical assessment were most prevalent prior to referral to the rheumatologist and whether targeted education could positively influence pattern recognition.

## Method

### Study design and participants

For the present study, qualitative outcome data collected alongside a prospective, controlled, multicentre, educational intervention study were used. The details of this study have been described elsewhere.^9^ In brief, GP-residents and their supervising GPs, recruited through the Department of General Practice from the Maastricht University Medical Centre (MUMC), were visited in two rounds by standardised patients (SPs) simulating axial SpA, peripheral SpA, or carpal tunnel syndrome (CTS), with an educational programme in between for part of the group. These SPs were healthy individuals trained to portray their case. All SPs were trained to simulate one case. Two 2-hour training sessions were organised and guided by several GPs and rheumatologists. SPs were trained in playing their role, and how to behave during the physical examination, in a realistically, valid, and reliable way. Close attention was paid on completion of the checklist to secure uniform data quality and comparability. Discrepancies in checklist rating scores were discussed and based on good reproducibility shown in previous studies, the authors assumed good representation of the cases by the SPs.^11–13^

**Figure 1.**
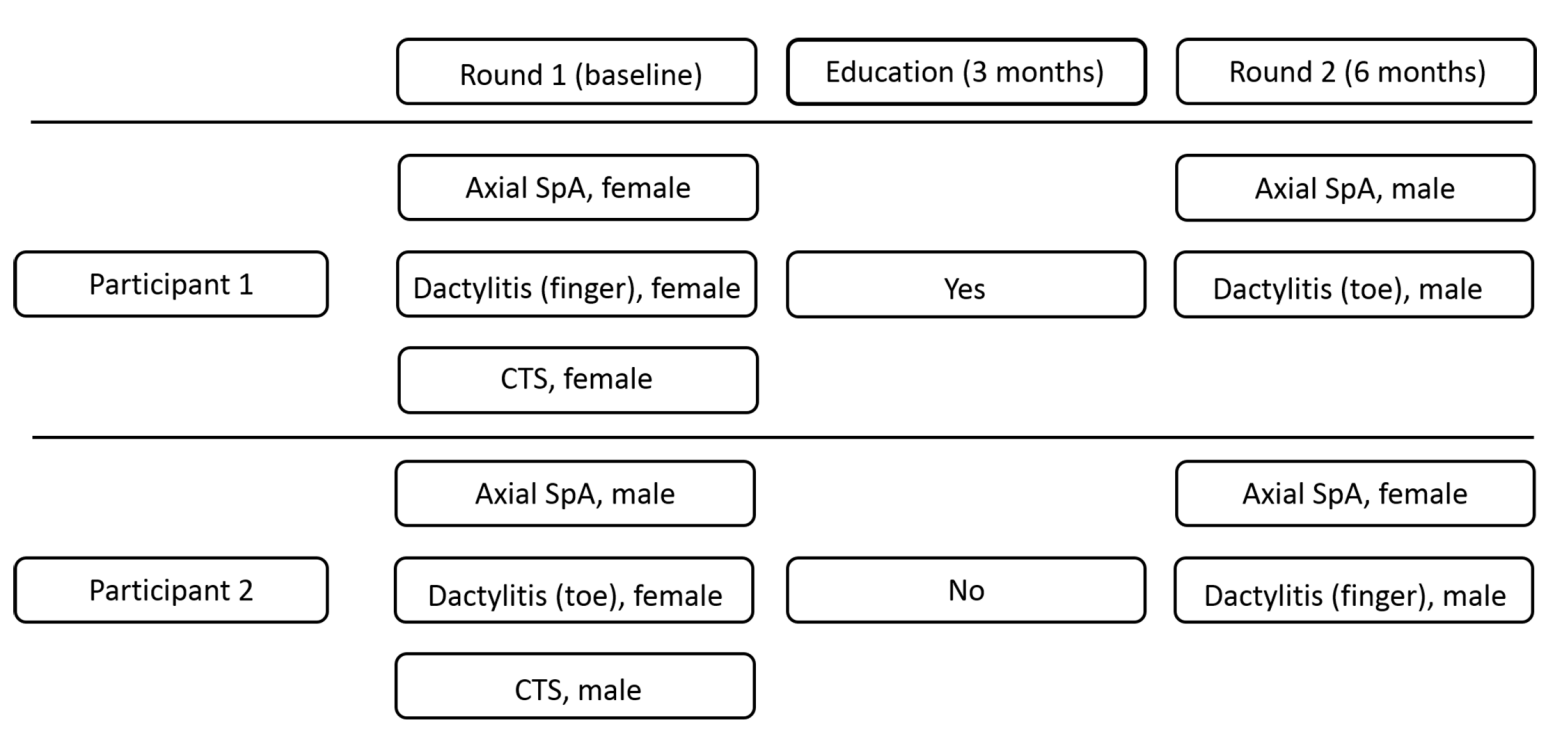
Example of a predefined schedule for 2 participants. CTS = carpal tunnel syndrome. SpA = spondyloarthritis.

Each GP(-resident) was visited by three SPs in round 1 (1 axial SpA case, 1 peripheral SpA case, and 1 CTS case) and two SPs in round 2 (1 axial SpA case and 1 peripheral SpA case). Each case was simulated by a male and a female SP, in random order, according to a predefined schedule (Figure 1).

Participants were informed that unannounced SPs would visit their practice but were unaware of the nature of the medical problem and study purpose. CTS was included as a diversionary tactic. The procedure that was followed during the visit of an SP who simulated CTS, was identical to the visit of an SP who simulated axial or peripheral SpA.

As part of the GP specialty training, GP-residents are assigned to a study group that receives education around a broad range of topics at the MUMC once per week. The study groups were alternately allocated to the intervention or control group. This allocation strategy was only applied to the GP-residents. After the first round of SP encounters, half of the included study groups received a 3-hour case-based educational programme on a regular training day (the intervention), without referring to the actual study. During this programme, among other topics, attention was paid to the concept and epidemiology of axial and peripheral SpA, history taking, physical examination, and criteria for referral. Special attention was paid to the extra-articular manifestations associated with SpA (such as, uveitis, psoriasis, and inflammatory bowel disease [IBD]), and family history of SpA. The remaining residents and all supervising GPs served as controls. The SP encounters took place 3 months before and 3 months after the educational intervention.

### Data collection

At the practice visit, the SPs identified themselves as an SP to the GP(-resident), without providing any further information. The SPs were instructed to behave as real patients and provide answers as they would in real clinical practice, to preserve a natural doctor–patient dialogue. After the consultation, the SPs immediately completed a case-specific checklist inquiring about relevant disease-related items (for example, presence of low back pain, swollen joints, or presence of extra-articular manifestations) and items on physical examination (for example, of the joints and/or back) that were discussed or dealt with by the GP(-resident).

On a separate form, the GP(-residents) had to indicate whether referral of the SP to another healthcare professional (and which) would be advised.

### Statistical analysis

Only participants that completed both rounds of SP encounters were included in the current analyses. Descriptive statistics were used to analyse demographic data.

The items listed on the case-specific checklist were categorised into five subgroups with questions regarding:

1. differentiation between a mechanical or inflammatory origin of the back pain or peripheral complaint (such as dactylitis);
2. presence of swollen joints, back pain or entheseal complaints;
3. presence of extra-articular manifestations;
4. family history; and
5. physical examination.

Differences in patterns of clinical assessment were explored for the education versus the control group and for participants who referred the SP correctly versus the participants who did not. Differences in referral were only further explored when at least 10 SPs in each group were available for in-depth comparison. Data were analysed by using descriptive statistics. SPSS software (version 22.0) and Excel^®^(version 2010) were used for all analyses.

## Results

### Participants

Sixty-eight subjects (38 GP-residents [32% male; mean age 28 years {standard deviation (SD) 1.6] and 30 GPs [53% male; mean age 52 years {SD} 5.9]) participated in the study. Three (4%) out of 68 GP(-residents) had a specific interest in musculoskeletal disorders (MSD). Both rounds of SP encounters were completed by 61 (90%) and 59 (87%) participants for the axial SpA and peripheral SpA cases, respectively. In the first round of SP encounters, the axial SpA cases were referred, or referral was considered, by one out of 18 (6%) participants in the intervention group and by four out of 43 (10%) participants in the control group. For the peripheral SpA case, the corresponding numbers were one out of 19 (5%) and two out of 40 (5%) for the intervention and control group, respectively. After the second round of SP encounters, a significantly higher proportion of GP-residents from the intervention group referred SPs to the rheumatologist as compared with the control group (change scores, axial SpA +71% versus +15% (*P*<0.01); peripheral SpA +48% versus 0% (*P *= 0.01).

### History taking: effect of education

#### Axial SpA

In round 1, about half of the GP(-residents) asked questions regarding the presence of inflammatory back pain, but almost none inquired about the presence of swollen joints, extra-articular manifestations, and/or a family history of SpA (Table 1). For instance, only two (11%) out of 18 GP-residents from the educational group inquired about the presence of past or present uveitis.

**Table 1.** Pattern of history taking of GP(-residents) facing a patient with axial SpA in round 1 and 2

Category	Item checked by GP or GP-resident	Educational group (*n* = 18)			Control group (*n* = 43)		
		Round 1	Round 2	Change score	Round 1	Round 2	Change score
1	Pain in lower back?	16 (89)	15 (83)	–1 (–6)	42 (98)	41 (95)	–1 (–2)
1	Night pain?	8 (44)	11 (61)	3 (17)	22 (51)	24 (56)	2 (5)
1	Morning stiffness?	10 (56)	17 (94)	7 (38)	16 (37)	33 (77)	17 (40)
1	Duration of morning stiffness?	8 (44)	13 (72)	5 (28)	11 (26)	19 (44)	8 (18)
1	Complaints worse or better over the day?	10 (56)	14 (78)	4 (22)	17 (40)	30 (70)	13 (30)
1	More complaints after resting?	11 (61)	16 (89)	5 (28)	17 (40)	15 (35)	–2 (–15)
1	Improvement on NSAID?	6 (33)	12 (67)	6 (34)	6 (14)	17 (40)	11 (26)
2	Swollen joints?	4 (22)	7 (39)	3 (17)	10 (23)	10 (23)	0 (0)
2	Entheseal complaints?	2 (11)	2 (11)	0 (0)	2 (5)	3 (7)	1 (2)
3	Uveitis?	2 (11)	8 (44)	6 (33)	3 (7)	3 (7)	0 (0)
3	Psoriasis?	0 (0)	5 (28)	5 (28)	1 (2)	2 (5)	1 (3)
3	IBD?	4 (22)	11 (61)	7 (39)	6 (14)	3 (7)	–3 (–7)
4	Family history AS?	4 (22)	10 (56)	6 (34)	7 (16)	12 (28)	5 (12)
4	Family history psoriasis?	0 (0)	5 (28)	5 (28)	0 (0)	2 (5)	2 (5)
4	Family history IBD?	2 (11)	8 (44)	6 (33)	2 (5)	1 (2)	–1 (3)

The values are expressed as number (percentage) of GP(-residents). Numbers may not add up due to rounding. AS = ankylosing spondylitis. IBD = inflammatory bowel disease. NSAID = non-steroidal anti-inflammatory drug. SpA = spondyloarthritis. Category: 1 = differentiation between a mechanical or inflammatory origin of the back pain; 2 = presence of swollen joints, back pain, or entheseal complaints; 3 = presence of extra-articular manifestations; 4 = family history.

In round 2, GP-residents who received the educational intervention were, as compared to round 1, more likely to ask questions about the presence of the inflammatory back pain. However, the most noticeable gain was seen in key questions regarding presence of extra-articular manifestations and a family history of SpA (Table 1).

In round 2, controls also tended to ask more questions regarding the presence of inflammatory back pain and family history of SpA. However, a clear increase in questions belonging to other subgroups (for example, extra-articular manifestations) was not observed (Table 1).

#### Peripheral SpA

In round 1, about 10% of the GP(-residents) inquired about the presence of extra-articular manifestations and 12% of GP(-residents) inquired about the presence of a positive family history of psoriasis (Table 2). Only one GP inquired about the presence of concomitant back pain.

**Table 2.** Pattern of history taking of GP(-residents) facing a patient with peripheral SpA in round 1 and 2

Category	Item checked by GP or GP-resident	Educational group (*n *= 19)			Control group (*n *= 40)		
		Round 1	Round 2	Change score	Round 1	Round 2	Change score
1	Duration of complaints?	19 (100)	18 (95)	–1 (–5)	40 (100)	39 (98)	–1 (–2)
1	Morning stiffness?	10 (53)	6 (32)	–4 (–21)	16 (40)	10 (25)	–6 (–15)
1	Duration of morning stiffness?	4 (21)	5 (26)	1 (5)	11 (28)	9 (23)	–2 (–5)
1	Complaints worse or better over the day?	12 (63)	5 (26)	–7 (–37)	15 (38)	15 (38)	0 (0)
1	More complaints after resting?	3 (16)	4 (21)	1 (5)	2 (5)	2 (5)	0 (0)
1	Improvement on NSAID?	16 (84)	15 (79)	–1 (–5)	29 (73)	22 (55)	–7 (–18)
2	Swollen joints?	9 (47)	16 (84)	7 (37)	29 (73)	35 (88)	6 (15)
2	Back pain?	0 (0)	6 (32)	6 (32)	1 (3)	4 (10)	3 (8)
2	Entheseal complaints?	0 (0)	1 (5)	1 (5)	3 (8)	4 (10)	1 (3)
2	Uveitis?	0 (0)	4 (21)	4 (21)	2 (5)	2 (5)	0 (0)
3	Psoriasis?	3 (16)	5 (26)	2 (20)	4 (10)	1 (3)	–3 (–8)
3	IBD?	2 (11)	6 (32)	4 (21)	6 (15)	5 (13)	–1 (3)
4	Family history AS?	0 (0)	1 (5)	1 (5)	0 (0)	0 (0)	0 (0)
4	Family history psoriasis?	1 (5)	5 (26)	4 (21)	3 (8)	0 (0)	–3 (–8)
4	Family history IBD?	0 (0)	4 (21)	4 (21)	2 (5)	2 (5)	0 (0)

The values are expressed as number (percentage) of GP(-residents). Numbers may not add up due to rounding. AS = ankylosing spondylitis. IBD = inflammatory bowel disease. NSAID = non-steroidal anti-inflammatory drug. SpA = spondyloarthritis. Category: 1 = differentiation between a mechanical or inflammatory origin of peripheral complaint; 2 = presence of swollen joints, back pain or entheseal complaints; 3 = presence of extra-articular manifestations; 4 = family history.

In round 2, GP-residents who received the educational intervention asked more questions regarding extra-articular complaints and family history of SpA. However, the observed gain was less evident as compared to the axial SpA case (Table 2).

### History taking: (considering) referral to the rheumatologist

Participants who considered referral or referred the SP with either axial or peripheral SpA in round 2, inquired more about the inflammatory character of the complaint, presence of extra-articular manifestations, and a positive family history of SpA as compared to participants who did not (consider) referral (Table 3). In round 2, 15 out the 19 (79%) participants for the axial SpA case and four of the five (80%) participants for the peripheral SpA case correctly diagnosed the SP and also referred the SP to the rheumatologist.

**Table 3.** Pattern of history taking in round 2: referral versus no referral

Axial spondyloarthritis				Peripheral spondyloarthritis			
Category	Item checked by GP or GP-resident	Referral yes (*n* = 25)	Referral no (*n* = 36)	Category	Item checked by GP or GP-resident	Referral yes (*n* = 12)	Referral no (*n* = 47)
1	Pain in lower back?	22 (88)	34 (94)	1	Duration of complaints?	12 (100)	45 (96)
1	Night pain?	19 (76)	16 (44)	1	Morning stiffness?	4 (33)	12 (26)
1	Morning stiffness?	24 (96)	26 (72)	1	Duration of morning stiffness?	4 (33)	10 (21)
1	Duration of morning stiffness?	17 (68)	15 (42)	1	Complaints worse or better over the day?	4 (33)	16 (34)
1	Complaints worse or better over the day?	21 (84)	23 (64)	1	More complaints after resting?	2 (17)	4 (9)
1	More complaints after resting?	10 (40)	12 (33)	1	Improvement on NSAID?	10 (83)	27 (57)
1	Improvement on NSAID?	15 (60)	14 (39)	2	Swollen joints?	11 (92)	40 (85)
2	Swollen joints?	9 (36)	8 (22)	2	Entheseal complaints?	1 (8)	4 (9)
2	Entheseal complaints?	2 (8)	3 (8)	2	Back pain?	6 (50)	4 (9)
3	Uveitis?	7 (28)	4 (11)	3	Uveitis?	5 (42)	1 (2)
3	Psoriasis?	6 (24)	1 (3)	3	Psoriasis?	4 (33)	2 (4)
3	IBD?	10 (40)	4 (11)	3	IBD?	5 (42)	6 (13)
4	Family history AS?	12 (48)	10 (28)	4	Family history AS?	1 (8)	0 (0)
4	Family history psoriasis?	6 (24)	1 (3)	4	Family history psoriasis?	4 (33)	1 (2)
4	Family history IBD?	6 (24)	3 (8)	4	Family history IBD?	2 (17)	4 (9)

The values are expressed as number (percentage) of GP (residents). AS = ankylosing spondylitis. IBD = inflammatory bowel disease. NSAID = non-steroidal anti-inflammatory drug. Category: 1 = differentiation between a mechanical or inflammatory origin of back pain or peripheral complaint; 2 = presence of swollen joints, back pain (peripheral SpA case) or entheseal complaints; 3 = presence of extra-articular manifestations; 4 = family history.

### Physical examination

When exploring the data of the physical examination, no clear pattern changes in both rounds of SP encounters could be identified (Tables 4 and 5; results for axial and peripheral SpA case before and after education). In round 1, the majority of GPs who faced an SP with either axial or peripheral SpA already performed an adequate physical examination of respectively the back and affected finger or toe. Lasgues sign was performed in almost half of the axial SpA cases; 16% of GP(-residents) performed the Schobers test during round 1.

**Table 4.** Pattern of physical examination of GP(-residents) facing a patient with axial and peripheral SpA in round 1 and 2

Item checked by GP or GP-resident	Educational group (*n* = 18)			Control group (*n *= 43)		
	Round 1	Round 2	Change score	Round 1	Round 2	Change score
Physical examination thoracic and lumbar spine • If yes, active flexion • If yes, lateroflexion • If yes, rotation	18 (100) 18 (100) 15 (83) 13 (72)	18 (100) 17 (94) 14 (78) 10 (56)	0 (0) –1 (–6) –1 (–6) –3 (–16)	38 (88) 37 (86) 29 (67) 19 (44)	37 (86) 37 (86) 27 (63) 18 (42)	–1 (–2) 0 (0) –2 (–4) –1 (–2)
Schober’s test	2 (11)	6 (33)	4 (22)	8 (19)	8 (19)	0 (0)
Occiput-to-wall distance	0 (0)	0 (0)	0 (0)	0 (0)	0 (0)	0 (0)
Chest mobility	0 (0)	0 (0)	0 (0)	0 (0)	1 (2)	1 (2)
Lasgue’s sign (straight leg raise)	10 (56)	8 (44)	–2 (–12)	20 (47)	20 (47)	0 (0)
Physical examination of the Achilles tendons	0 (0)	0 (0)	0 (0)	1 (2)	1 (2)	0 (0)
Physical examination of peripheral joint	0 (0)	0 (0)	0 (0)	2 (5)	2 (5)	0 (0)

The values are expressed as number (percentage) of GP(-residents). Numbers may not add up due to rounding.

**Table 5.** Pattern of physical examination of GP(-residents) facing a patient with peripheral SpA in round 1 and 2

Item checked by GP or GP-resident	Educational group (*n* = 19)			Control group (*n *= 40)		
	Round 1	Round 2	Change score	Round 1	Round 2	Change score
Range of motion affected finger or toe	12 (63)	10 (53)	–2 (–11)	22 (55)	14 (35)	–8 (–20)
Range of motion wrist or ankle of affected limb	1 (5)	3 (16)	2 (11)	2 (5)	1 (3)	–1 (–2)
Physical examination on both hands or feet	6 (32)	6 (32)	0 (0)	12 (30)	6 (15)	–6 (–15)
Palpation joints hands or feet	16 (84)	11 (58)	–5 (–26)	23 (58)	26 (65)	3 (8)
Palpation MCP or MTP joint affected finger or toe	14 (74)	11 (58)	–3 (–16)	16 (40)	22 (55)	6 (15)
Palpation other MCP or MTP joints	5 (26)	6 (32)	1 (5)	9 (23)	10 (25)	1 (2)
Palpation PIP joint affected finger or toe	13 (68)	11 (58)	–2 (–10)	22 (55)	24 (60)	2 (5)
Palpation other PIP joints	4 (21)	3 (16)	–1 (–5)	8 (20)	9 (23)	1 (2)
Palpation DIP joint affected finger or toe	14 (74)	11 (58)	–3 (–16)	21 (53)	22 (55)	1 (2)
Palpation other DIP joints	5 (26)	3 (16)	–2 (–10)	6 (15)	7 (17)	1 (2)
Physical examination hands (in case of dactylitis foot) and vice versa	0 (0)	4 (21)	4 (21)	1 (3)	3 (8)	2 (5)
Physical examination Achilles tendons	0 (0)	0 (0)	0 (0)	0 (0)	3 (8)	3 (8)
Physical examination other joints	0 (0)	0 (0)	0 (0)	0 (0)	2 (5)	2 (5)

The values are expressed as number (percentage) of GP(-residents). Numbers may not add up due to rounding. MCP = metacarpophalangeal. MTP = metatarsophalangeal. PIP = proximal interphalangeal.

## Discussion

### Summary

In the present study, patterns in clinical assessment of patients suspected of having SpA were identified that were most prevalent prior to correct referral to the rheumatologist. About half of the participants asked at least one question to differentiate between inflammatory and mechanical back pain or peripheral complaints in the first round of SP encounters. Almost none of the participants asked questions that are critical for pattern recognition, such as presence of extra-articular manifestations and family history, in round 1. However, recognition of the clinical pattern suggestive of SpA was important for referral of the SP to the rheumatologist. This study showed that education positively influenced the abilities of the participating GP(-residents) to recognise patterns suggestive of SpA.

### Strengths and limitations

A strength of this study was its prospective, multicentre design. In addition, a control group was included for the evaluation of the effect of education. Some limitations however need to be acknowledged. First, the SPs did not truly have SpA, but simulated the disease. In the peripheral SpA case, abnormalities were only visible on a photograph. GP(-residents) may therefore have omitted certain questions during history taking or did not perform a more thorough physical examination. Despite this, more than half of the GP(-residents) palpated the affected finger or toe during physical examination. Second, the GP(-residents) were all living in the southern region of the Netherlands. This may limit generalisability to a wider population and reproducibility. However, the main goal of this study was not to extrapolate the current findings to all GPs, but to explore deficiencies in pattern recognition that probably need attention in future educational programmes. Third, only the study groups of GP-residents were randomly assigned to the intervention or control group. All GPs were included in the control group. This may also limit generalisability to a wider population since the effectiveness of the intervention in more senior GPs is unknown. Last, it could be argued that recognition and referral of SpA in the present study is not solely based on improved history taking. Instead, the educational intervention about SpA and subsequent visit of an SP may already raise suspicion on a diagnosis of SpA. In this case, the participants who received the educational intervention may have asked more questions in the direction of SpA purely on the basis of their participation in a study. However, the participants did not know that education was part of the study and therefore the current results reflect the genuine performance of GP(-residents) in daily practice.

### Comparison with existing literature

Recognition of what is abnormal and what needs referral to secondary care requires good clinical knowledge and awareness. Earlier studies have shown that GPs lack confidence regarding the clinical assessment of patients with MSD, often due to limited education about MSD during their GP training.^14–16^ This important omission may in turn lead to lack of interest in rheumatic disorders/MSD, despite their high prevalence in primary care. In this study, only three (4%) of the 68 GP(-residents) had a specific interest in MSD; interestingly, in none of the cases from these three GP(-residents), were the SPs referred to the rheumatologist.

The present study indicates that education is an important opportunity for improvement of history taking and raising awareness about SpA by GPs. The most noticeable gains were seen in a higher frequency of questions asked regarding extra-articular manifestations and family history of SpA. The fact that special attention was paid to these important components of history taking during the educational intervention, may explain this effect.

Physical examination was not clearly influenced by the educational intervention and was not an important factor for referral of the SP to the rheumatologist. For the axial SpA patient, physical examination of the back early in the disease course is of limited diagnostic value. The spinal mobility measurements as well as posture may still be normal.^17^ However, in peripheral SpA, physical examination of the peripheral joints could contribute to correctly recognise the pattern and hence referral to the rheumatologist.

### Implications for practice

In the cases, as simulated by the SPs in this study, considering referral of the SP to secondary care was defined as the most appropriate option. On request, the SPs described an inflammatory pattern of the complaint and the family history was positive for extra-articular manifestations of SpA. Psychosocial factors such as negative work perceptions were absent. Despite the clear-cut nature of the simulated case, <10% of GP(-residents) referred the patient. Pattern recognition of patients suspected for SpA is essential for referral to a rheumatologist. The present study shows that targeted education can help GPs to improve their history taking. Focusing on history taking alone already increased the chance of referral to the rheumatologist and should therefore be offered to all GPs in order to successfully implement a referral strategy in primary care.
